# The relationship between polycystic ovary syndrome and coronary heart disease: a bibliometric analysis

**DOI:** 10.3389/fendo.2023.1172750

**Published:** 2023-05-08

**Authors:** Xuzhi Liang, Haijing He, Hao Zeng, Liuyi Wei, Jiahuang Yang, Yuqi Wen, Siqi Fan, Jiangtao Fan

**Affiliations:** ^1^ Department of Gynecology, Guangxi Medical University First Affiliated Hospital, Nanning, Guangxi, China; ^2^ Department of Ophthalmology, University of Bonn, Bonn, North Rhin-Westphalia, Germany

**Keywords:** polycystic ovary syndrome, coronary heart disease, bibliometric analysis, risk factor, VOSviewer, citespace

## Abstract

**Background:**

Polycystic ovary syndrome (PCOS) is one of the most common gynecological endocrine diseases for women of puberty and reproductive age. PCOS can affect women’s health for the rest of their lives since the incidence of coronary heart disease (CHD) may increase in the perimenopausal and senile periods among PCOS women compared with non-PCOS women.

**Method:**

A literature retrieval based on the Science Citation Index Expanded (SCI-E) database. All obtained records results were downloaded in plain text format for subsequent analysis. VOSviewer v1.6.10, Citespace and Microsoft Excel 2010 software were utilized for analyzing the following terms: countries, institutions, authors, journals, references and keywords.

**Results:**

There were 312 articles retrieved from January 1, 2000 to February 8, 2023, and the frequency of citations was 23,587. The United States, England, and Italy contributed the majority of the records. Harvard University, the University of Athens, and Monash University were the top 3 most productive institutions with publications on the relationship between PCOS and CHD. Journal of clinical endocrinology & metabolism ranked first with the highest publications (24 records), followed by Fertility and sterility (18 records). The keywords were divided into six clusters in the overlay keywords network: (1) the correlation between CHD risk factors and PCOS women; (2) the relationship between cardiovascular disease and female reproductive system hormone secretion; (3) the interaction between CHD and metabolic syndrome; (4) the relationship between c-reactive protein and endothelial function and oxidative stress in PCOS patients; (5) the potential positive effect of metformin on reducing CHD risk factors in PCOS patients; (6) the study of serum cholesterol and body-fat distribution in patients with CHD in PCOS. Oxidative stress, genome-wide association, obesity, primary prevention, and sex difference were main hotspots in this field in recent five years according to the keyword citation burst analysis.

**Conclusion:**

The article obtained the hotspots and trends and provided a reference for subsequent research on the association between PCOS and CHD. Moreover, it is hypothesized that oxidative stress and genome-wide association were frontier hotspots in studies that explore the relationship between PCOS and CHD, and prevention research may be valued in the future.

## 1 Introduction

Polycystic ovary syndrome (PCOS) is one of the most common gynecological endocrine diseases in adolescent and reproductive-age women, with an incidence of 6-10% (1, 2). Its clinical manifestations are varied and mainly characterized by irregular menstruation, anovulation or high androgen levels, infertility, and an increased risk of metabolic diseases (3). The risk of hypertension in PCOS women is twice that in non-PCOS women, which may be related to insulin resistance or hyperinsulinemia that damages vascular smooth muscle cells (VSMCs) and leads to the thickening and decreased elasticity of vascular walls (4). PCOS patients are prone to complications such as hypertension, abnormal lipid metabolism, type 2 diabetes mellitus (T2DM) and obesity, which are risk factors for coronary heart disease (CHD) (5). PCOS could enhance the overall cardiovascular risk, especially myocardial infarction, angina pectoris, and revascularization (6, 7). Increased serum triglyceride and low-density lipoprotein cholesterol (LDL-C) levels, decreased high-density lipoprotein cholesterol (HDL-C) cholesterol levels, and the altered ratio of apolipoprotein B to apolipoprotein A1 levels in PCOS patients are also associated with increased risk of CHD. In addition, high levels of C-reactive protein (CRP) and homocysteine in PCOS patients have been considered risk factors for the development of CHD (8, 9).

However, after searching various major databases, we found considerable literature on the relationship between the risk of CHD and PCOS. Although some scholars have done literature review studies (10–12), the studies’ systematization and comprehensive visual analysis still need to be improved.

Bibliometric analysis is a method of analyzing, cleaning and mining the quantitative information of literature using mathematical and statistical functions, which explore structures, characteristics and laws of science and technology. The VOSviewer software developed by Van Eck of Netherlands University can intuitively display terms in different color clusters and clearly show the connections between clusters (13). CiteSpace software is a visual tool developed by Chen Chaomei that can realize keyword measurement and literature data analysis, which integrates cluster analysis and social network analysis. Also, both are important tools for mining research hotspots in a certain field and predicting development trends (14).

Science Citation Index Expanded (SCI-E) database, as one of the three major databases in the core collection of Web of Science (WOS) databse, is an authoritative and high-impact citation index database of scientific journals. Therefore, this study intended to use VOSviewer and CiteSpace to visually analyze the literature related to the relationship between CHD and PCOS in the SCI-E database to explore the main research content and track the research hotspots. Based on the data analysis findings in the literature, we attempted to describe the significant impact of PCOS on the health integrity of patients in order to provide a reference for the further research direction of the relationship between PCOS and CHD and the preventive measures for PCOS patients with CHD.

## 2 Methods

### 2.1 Data source and search strategy

Our study referred to the general internationally accepted method of bibliometrics. The SCI-E database (http://www.webofscience.com/), the most common database for bibliometrics, was used to retrieve literature. The retrieval method was subject-term searching. Using keywords related to polycystic ovary syndrome, including “polycystic ovary syndrome”, “polycystic ovarian syndrome”, “PCOS”, and “Stein-Leventhal Syndrome”; and coronary heart disease-related keywords, including “coronary heart disease”, “CHD”, and “Coronary Diseases”, and operation method was “AND”. All obtained records results were downloaded in plain text format for subsequent analysis. We did all the searches and data exports on the same day (February 8, 2023) since metrics constantly changed over time.

### 2.2 Inclusion and exclusion criteria

The inclusion and exclusion of studies were based on the filters of the WOS database. The studies that met the following criteria were included (1): articles published in the period from January 1, 2000 to February 8, 2023 (2), articles about PCOS and CHD, (3) original articles or reviews, and (4) published in English. Exclusion criteria were (1) repeated publications, (2) proceeding paper, editorial material, meeting abstract, book chapters, letters, early access, correction, reprint, and other types. Two researchers strictly screened the literature by detailed examining the abstract section according to the inclusion and exclusion criteria. The third researcher decided on the contents with differences and uncertainties.

### 2.3 Data collection and cleaning

The following basic information was collected for each article: countries, authors, institutions, journals, references and keywords. Keywords with the same meaning but in different styles were standardized: “syndrome pcos” was replaced by “polycystic ovary syndrome”, “coronary-artery disease” was replaced by “coronary heart disease”, “type 2 diabetes mellitus” was replaced by “type 2 diabetes”, “obese women” and “overweight” were replaced by “obesity”, “BMI” was replaced by “body mass index”.

### 2.4 Bibliometric software

The above plain text was imported into VOSviewer v.1.6.10 (13) and CiteSpace 5.5.R2 (14) software. VOSviewer (http://www.vosviewer.com) is software for mapping scientific knowledge, which can construct and visualize the relationship between network data. It shows the structure, evolution and cooperation of the knowledge domain with its outstanding feature of the ability of graphic display and large-scale data analysis. Compared with VOSviewer, CiteSpace software has its certain advantages in visualizing burst words and revealing the dynamic development and change of research hotspots and discipline.

### 2.5 Data analysis

In the knowledge map generated by the VOSviewer, research projects were presented, including countries, institutions, authors, co-cited references and keywords, et al. The research projects can be demonstrated as nodes, and links between nodes represent collaboration, co-occurrence or co-citations among them. The bibliographic co-citation analysis and keyword co-occurrence analysis networks were used to construct a knowledge map of studies on the association between PCOS and CHD. Reference co-citation cluster analysis can summarize the main topics in the research field. Keyword co-occurrence analysis can illustrate the theme of the literature. The distribution trend of obtained research was generated by Microsoft Excel 2010 software. The burst words were analyzed by CiteSpace.

## 3 Results

### 3.1 Search results

**Figure 1:**
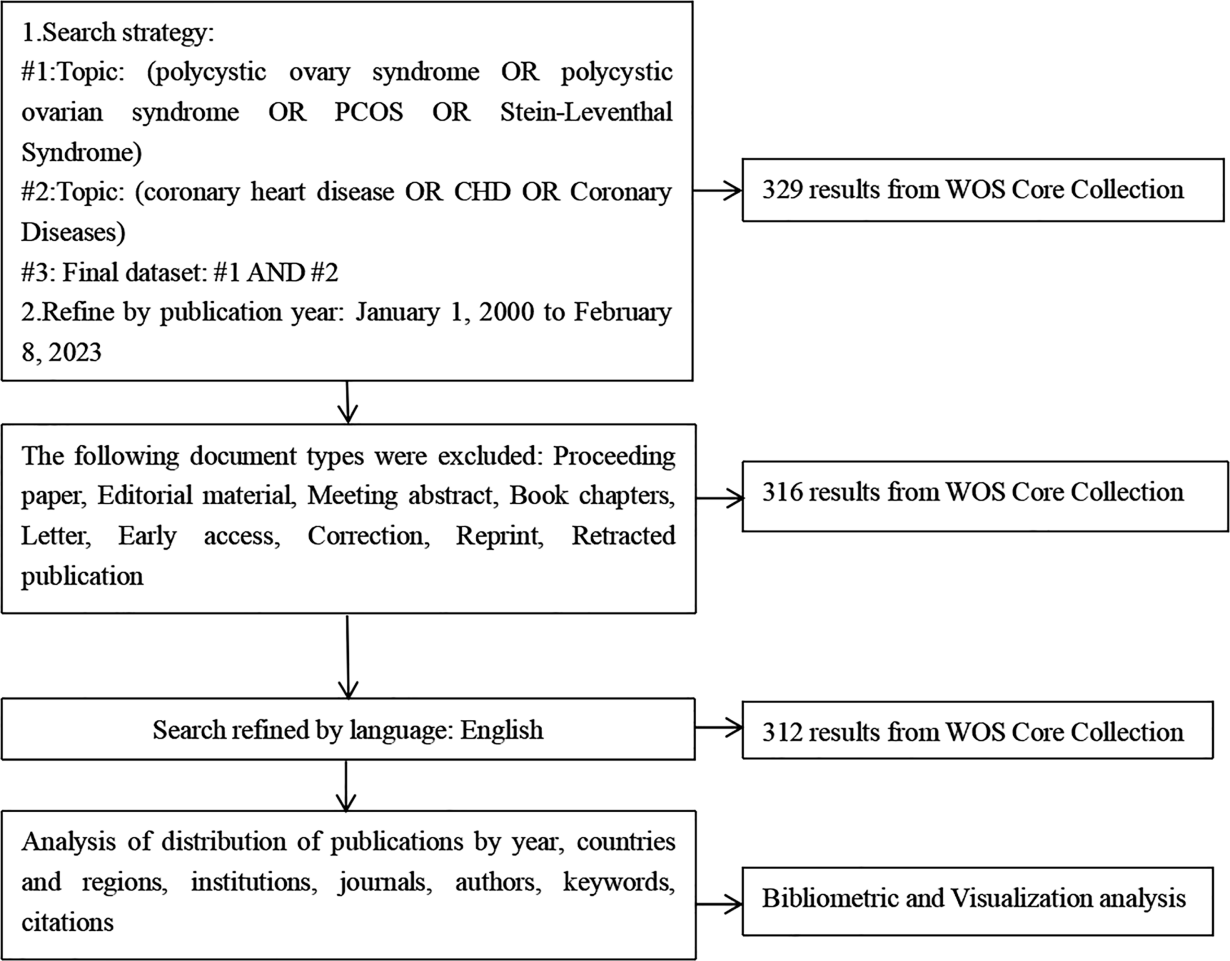
A frame flow diagram. The diagram shows detailed selection criteria for POI therapy publications from the WOS database and the steps of the bibliometric analysis.

**Figure 2:**
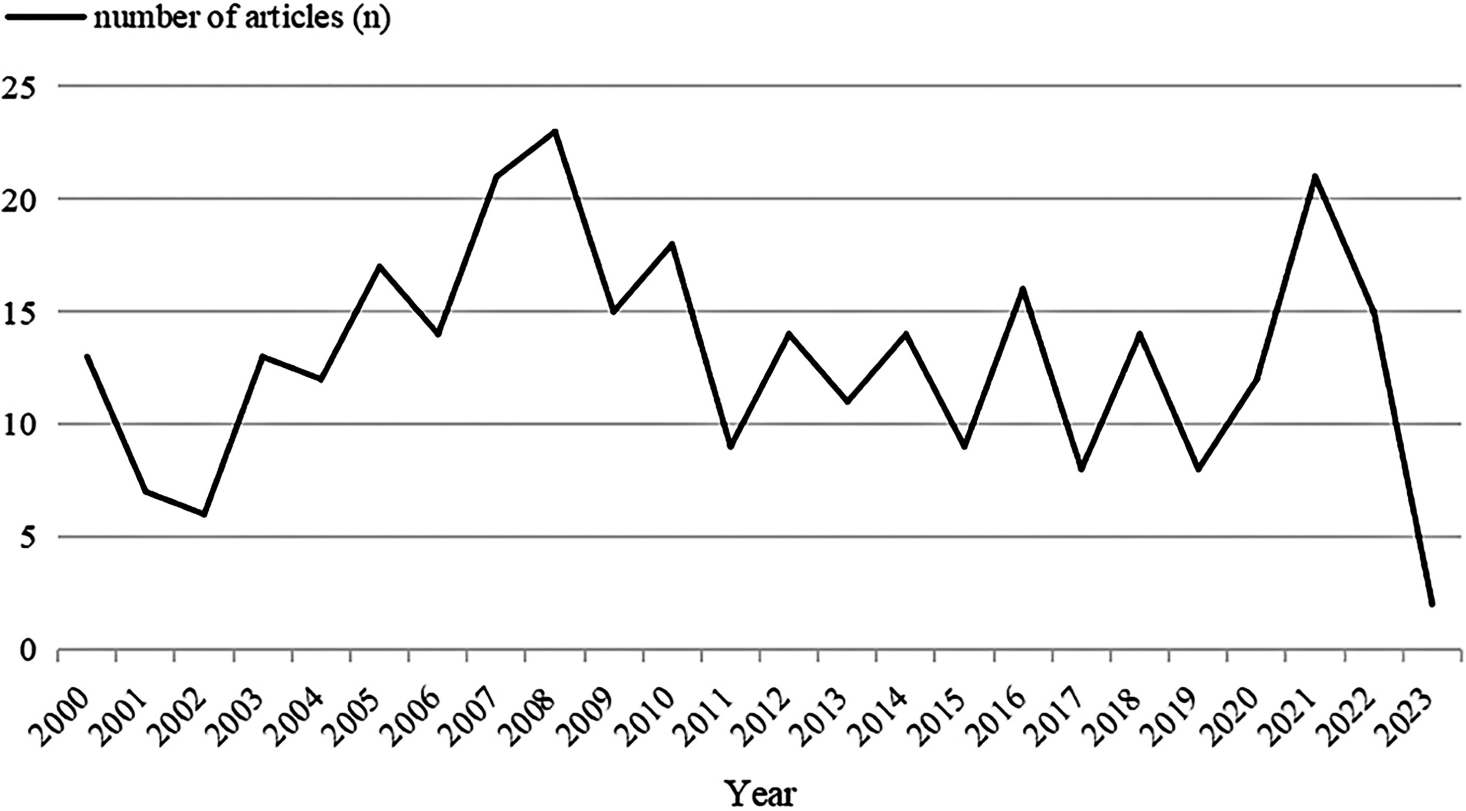
The distribution trend from January 1, 2000 to February 8, 2023.

There were a total of 312 records in the search results from January 1, 2000 to February 8, 2023 (**Figure 1**). More than half of the records were original articles (202/312, 64.7%), which greatly reflected the development trends and changes in the field of research on the association between CHD and PCOS. The distribution of publications by year was shown in **Figure 2**, demonstrating a flat trend. As of February 8, 2023, there are only two articles, but this number will continue to increase.

### 3.2 Analysis of author’s country of origin

**Figure 3:**
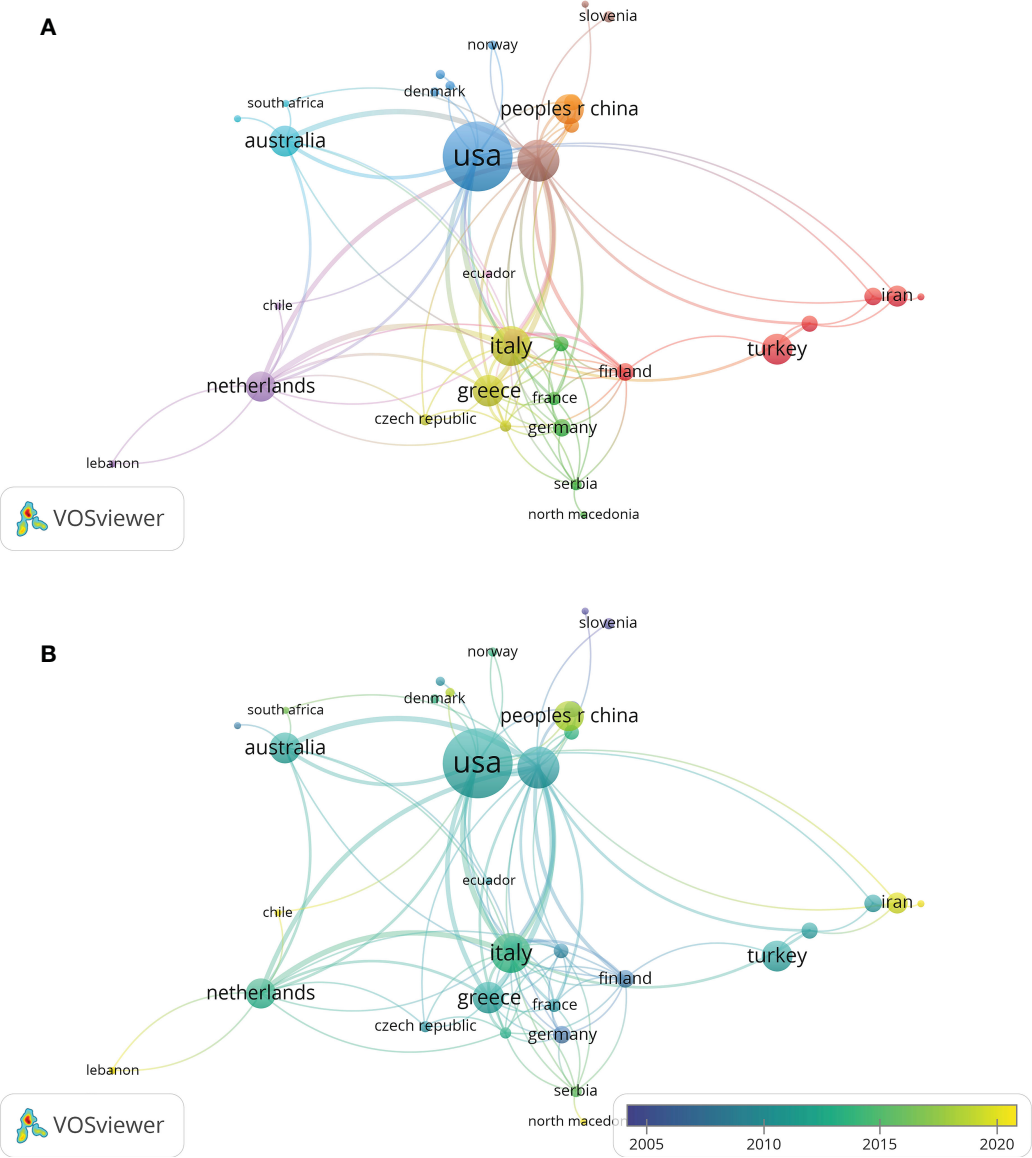
A visualization map of countries. There are nodes (circles) in the figure, representing countries. The larger the circle, the more documents were produced by that country. A connection between 2 nodes means that 2 countries appear in a document simultaneously; that is, 2 countries have a cooperative relationship in this document. **(A)** Network diagram of the top 35 countries. **(B)** Dynamics and trends of the top 35.

According to the search results, 312 articles were from 46 countries, and the top 35 countries by the number of publications were presented in **Figure 3**. The top 10 countries engaged in studies on the association between PCOS and CHD were shown in **Table 1**. The United States of America (USA) had the largest number of publications (n=105), followed by England (n = 38) and Italy (n = 34). According to the citation analysis, there were 10,198 citations in the United States, followed by England (4,373 citations) and Australia (2,959 citations).

**Table 1:** Top 10 most productive countries/regions with publications on the relationship of PCOS and CHD from 2000 to 2023.

Rank	Counties/regions	Documents (n)	Citations (n)	Average citations (n)	Total link strength
1	USA	105	10198	97.1238	23
2	England	38	4373	115.0789	25
3	Italy	34	1464	43.0588	15
4	Greece	22	1629	74.0455	9
5	Australia	21	2590	123.3333	8
6	Turkey	21	687	32.7143	2
7	Netherlands	20	1475	73.75	9
8	China	20	450	22.5	3
9	Iran	10	162	16.2	4
10	Spain	8	646	80.75	5

### 3.3 Universities and institutions

A total of 513 institutions were involved in these articles. **Table 2** showed the top 15 institutions with more than five related publications. Among them, 8/15 were from the USA, 3/15 were from Greece, and 2/15 were from England. Harvard University was the top with the most publications, followed by the University of Athens, Monash University, Aristotle University of Thessaloniki, and Brigham & Women’s Hospital. The top 15 institutions published 100 articles, accounting for 32.15% of the total. The University of Glasgow achieved the highest citations.

**Table 2:** Top 15 institutions with publications on the relationship of PCOS and CHD from 2000 to 2023.

Rank	Institution	Country/ regions	Documents (n)	Citations (n)	Average citations (n)	Total link strength
1	Harvard University	USA	12	1387	116	47
2	University of Athens	Greece	10	1037	104	18
3	Monash University	Australia	9	1449	161	24
4	Aristotle University of Thessaloniki	Greece	8	464	58	7
5	Brigham & Womens Hospital	USA	8	781	98	23
6	Baylor College of Medicine	USA	6	1017	170	40
7	University of Glasgow	England	6	1937	323	7
8	University Medical Center Utrecht	Netherlands	6	481	80	15
9	Magna Graecia University Of Catanzaro	Greece	5	325	65	12
10	The Pennsylvania State University	USA	5	309	62	32
11	University College London	England	5	693	139	4
12	The University of Alabama at Birmingham	USA	5	431	86	22
13	University of California, San Francisco	USA	5	365	73	28
14	University of Pittsburgh	USA	5	483	97	15
15	Virginia Commonwealth University	USA	5	582	116	27

### 3.4 Analysis of author cooperation

**Figure 4:**
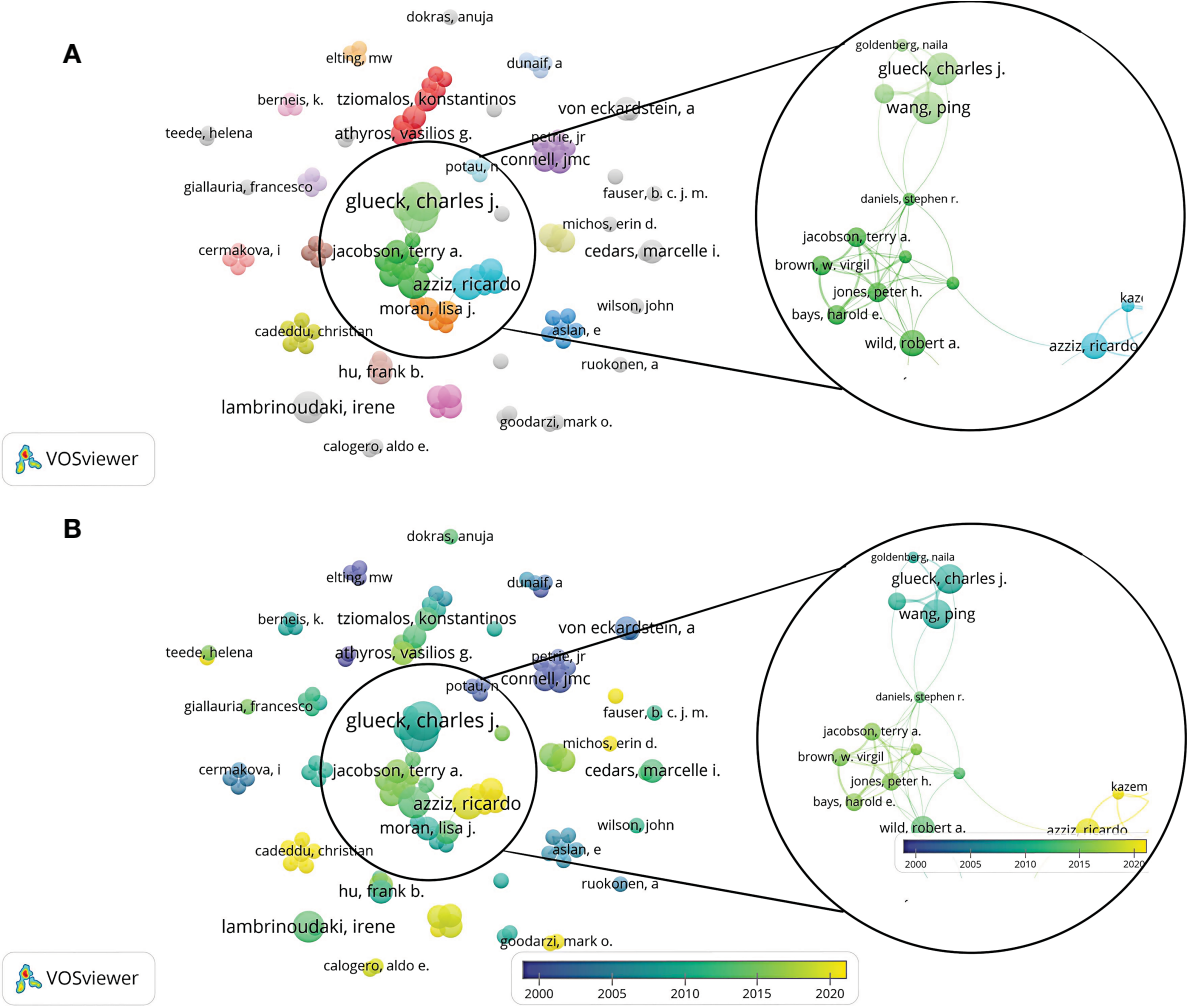
A visualization map of authors. There are nodes (circles) in the figure, representing authors. The larger the circle, the more documents produced by that author. A connection between 2 nodes means that 2 authors appear in a document simultaneously; that is, 2 authors have a cooperative relationship in this document. **(A)** Network diagram of the authors with a frequency ≥ 2 times. **(B)** Dynamics and trends of the authors with a frequency ≥ 2 times.

A total of 1,518 authors were involved in related publications, authors with a frequency ≥ 2 times were incorporated in the collaboration network analysis, and 111 authors were obtained. Dr. Wang, Ping, Dr. Glueck, Charles J, Dr. Wild, Robert A, Dr. Lambrinoudaki, Irene, Dr. Azziz, and Dr. Ricardo had the highest number of publications. They were distributed in different clusters, showing close cooperative relationships with other authors (**Figure 4**). Although Dr. Wild, Robert A ranked non-first in the number of articles published, Dr. Wild, Robert A’s group was at the center of the network, working tightly with other groups. In addition, Dr. Lambrinoudaki Irene was not associated with the center’s collaboration network and had less collaboration with other authors.

### 3.5 Distribution of publications by journals

A total of 170 journals were included in related research. The top 11 journals with more than five publications were shown in **Table 3**. Over the past 23 years, the Journal of clinical endocrinology & metabolism (24 records) showed the highest number of publications, followed by Fertility and sterility (18 records) and Human reproduction (11 records). In addition, the 2021 impact factor (IF) ranged from 2.28 (Gynecological Endocrinology) to 17.18 (Human reproduction update). The 5-year IF ranged from 2.24 (Gynecological Endocrinology) to 19.42 (Human reproduction update). Moreover, the Journal of Clinical Endocrinology and Metabolism represented the highest H-index, indicating the higher quality of studies published in this journal.

**Table 3:** Top 11 journals by the number of publications on the relationship of PCOS and CHD from 2000 to 2023.

Rank	Journals	Documents (n)	Citations (n)	Average citations (n)	Total link strength	2021 IF	5-year IF	H-index
1	Journal of clinical endocrinology & metabolism	24	2880	120	92	6.13	6.83	363
2	Fertility and sterility	18	996	55	36	7.49	8.11	217
3	Human reproduction	11	758	67	27	6.35	7.74	236
4	Clinical endocrinology	7	629	90	39	3.52	3.82	153
5	Current pharmaceutical design	6	100	17	6	3.31	3.55	166
6	European journal of endocrinology	6	293	49	13	6.56	6.81	155
7	Gynecological endocrinology	6	219	37	5	2.28	2.24	65
8	Human reproduction update	6	1307	218	32	17.18	19.42	190
9	Metabolism-clinical and experimental	6	286	48	19	13.93	10.86	145
10	Journal of endocrinological investigation	5	176	35	15	5.47	4.63	87
11	Maturitas	5	138	28	10	5.11	5.52	105

### 3.6 Top 10 citation publications

Of the 312 records, the top 10 publications ranked by citation were listed in **Table 4**. The most cited article was published in Endocrine Reviews by Frank W Booth et al. in 2012 (15) with 1201 citations, which was higher than that of the second paper (955 citations) (16). The third (17) and fourth (18) cited papers were reviews, with 670 and 523 citations, respectively. The fifth article by Wild S, Pierpoint T et al. (19) conducted a long-term follow-up in women diagnosed with PCOS in the United Kingdom before 1979, which concluded that a history of non-fatal cerebrovascular disease and cardiovascular risk factors, including diabetes, were more common in PCOS women compared with non-PCOS women.

**Table 4:** Top 10 citation publications.

Rank	Authors	Number of citations (n)	Titles	Journals
1	Booth (2012)	1201	Lack of exercise is a major cause of chronic diseases	Comprehensive physiology
2	Pedersen (2015)	955	Exercise as medicine - evidence for prescribing exercise as therapy in 26 different chronic diseases	Scandinavian journal of medicine & science in sports
3	Moran (2010)	670	Impaired glucose tolerance, type 2 diabetes and metabolic syndrome in polycystic ovary syndrome: a systematic review and meta-analysis	Human reproduction update
4	Liu (2003)	523	Androgens and cardiovascular disease	Endocrine reviews
5	Wild (2000)	469	Cardiovascular disease in women with polycystic ovary syndrome at long-term follow-up: a retrospective cohort study	Clinical endocrinology
6	Wu (2003)	440	Androgens and coronary artery disease	Endocrine reviews
7	Garvey (2016)	393	American association of clinical endocrinologists and american college of endocrinology comprehensive clinical practice guidelines for medical care of patients with obesity	Endocrine practice
8	Talbott (2000)	382	Evidence for association between polycystic ovary syndrome and premature carotid atherosclerosis in middle-aged women	Arteriosclerosis thrombosis and vascular biology
9	Samson (2014)	338	Metabolic syndrome	Endocrinology and metabolism clinics of north america
10	Solomon (2002)	338	Menstrual cycle irregularity and risk for future cardiovascular disease	Journal of clinical endocrinology & metabolism

### 3.7 Co-citation distribution

**Figure 5:**
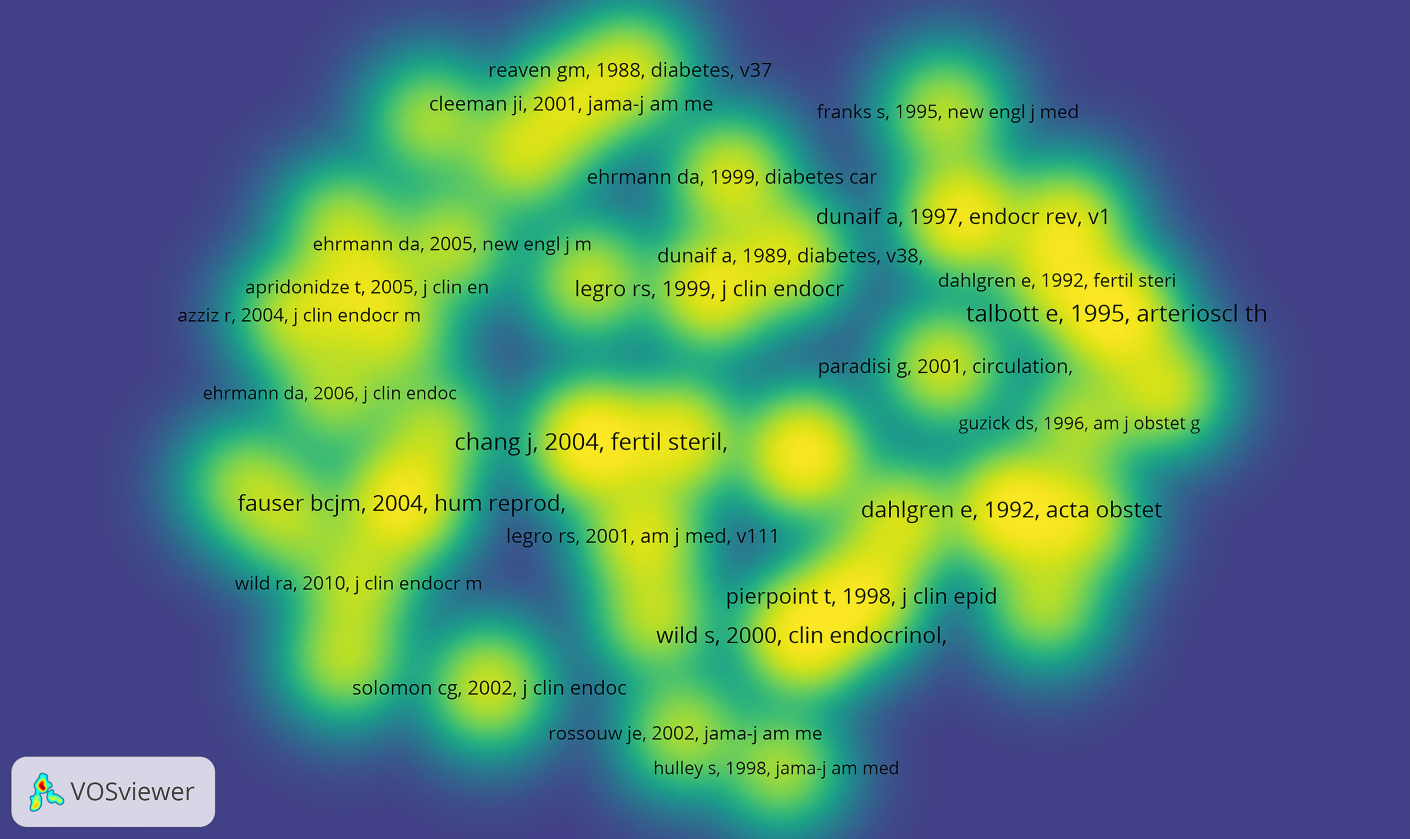
Network map of co-citation references.

**Figure 6:**
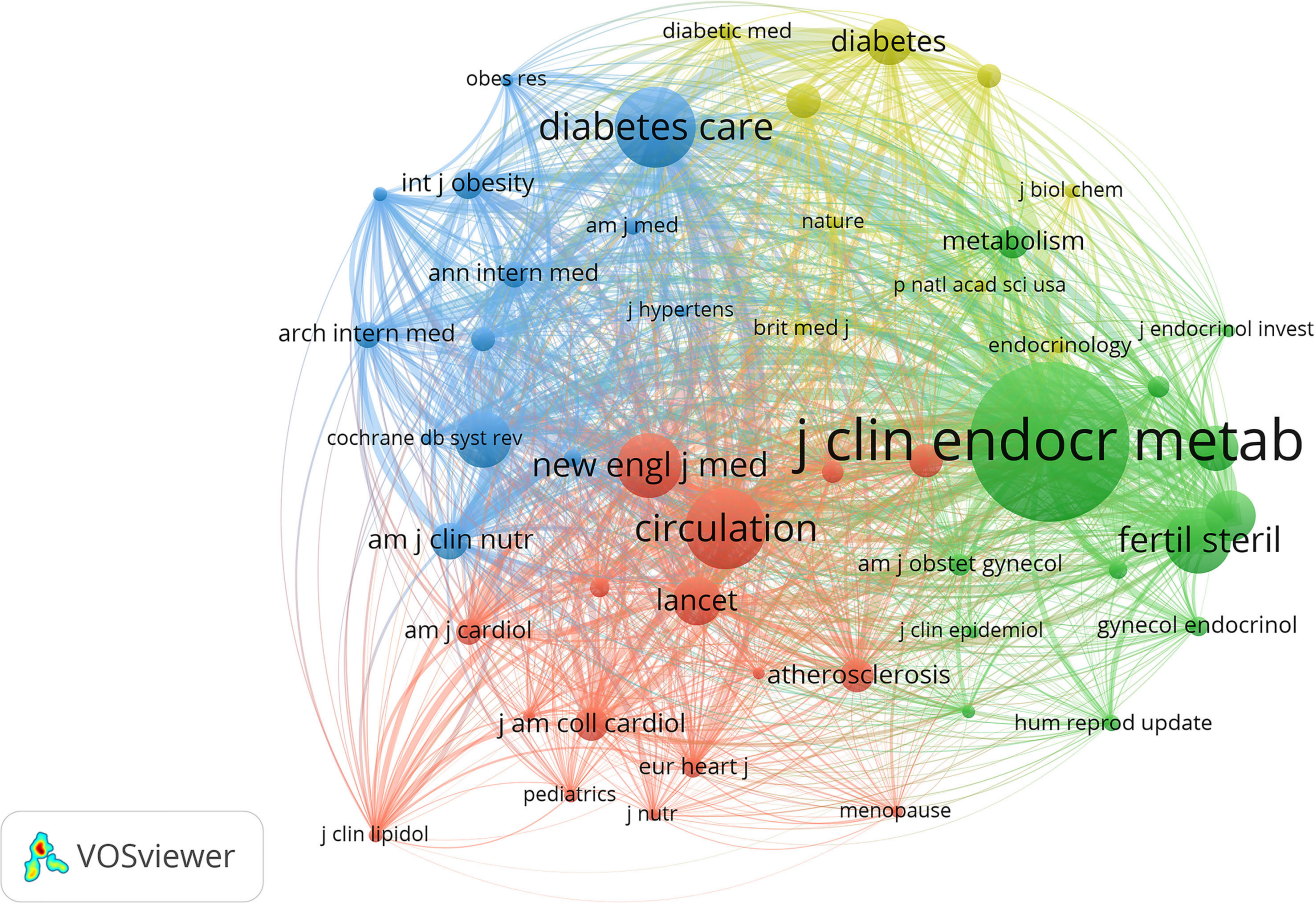
VOSviewer visualization map of most commonly cited journals. Of the 3,227 journals of cited references, the minimum number of citations for a single journal was set at 100; by analyzing the citation number of the journals, 51 journals reached the threshold.

Through the co-citation analysis of the cited documents of related research, the research foundation in this field can be effectively constructed. Of the 23,587 cited references, the minimum number of citations for a single document was set at 20. By analyzing the citation frequency of 23,587 documents, 42 documents reached the threshold (**Figure 5**). The size of the node corresponds to the frequency of references. Analysis of the top 10 citation publications was represented in **Table 5**. Of the 3227 journals of cited references, the minimum number of citations for a single journal was set at 100. By analyzing the citation number of the journals, 51 journals reached the threshold (**Figure 6**). The size of the node corresponds to the number of cited references.

**Table 5:** Top 10 co-citations.

Rank	Authors	Number of citations (n)	Titles	Journals
1	Chang (2004)	51	Revised 2003 consensus on diagnostic criteria and long-term health risks related to polycystic ovary syndrome	Fertility and sterility
2	Talbott (1995)	48	Coronary heart disease risk factors in women with polycystic ovary syndrome	Arteriosclerosis, Thrombosis, and Vascular Biology
3	Talbott (2000)	46	Evidence for association between polycystic ovary syndrome and premature carotid atherosclerosis in middle-aged women	Arteriosclerosis, Thrombosis, and Vascular Biology
4	Dahlgren (1992)	45	Polycystic ovary syndrome and risk for myocardial infarction. Evaluated from a risk factor model based on a prospective population study of women	Acta obstetricia et gynecologica scandinavica
5	Wild (2000)	45	Cardiovascular disease in women with polycystic ovary syndrome at long-term follow-up: a retrospective cohort study	Clinical endocrinology
6	Fauser (2004)	43	Revised 2003 consensus on diagnostic criteria and long-term health risks related to polycystic ovary syndrome (PCOS)	Human reproduction
7	Dunaif (1997)	42	Insulin resistance and the polycystic ovary syndrome: mechanism and implications for pathogenesis	Endocrine reviews
8	Pierpoint (2000)	39	Mortality of women with polycystic ovary syndrome at long-term follow-up	Journal of clinical epidemiology
9	Legro (2014)	38	Prevalence and predictors of risk for type 2 diabetes mellitus and impaired glucose tolerance in polycystic ovary syndrome: a prospective, controlled study in 254 affected women	The journal of clinical endocrinology & metabolism
10	Matthews (2002)	37	Homeostasis model assessment: insulin resistance and beta-cell function from fasting plasma glucose and insulin concentrations in man	Diabetologia

### 3.8 Analysis of keywords

**Figure 7:**
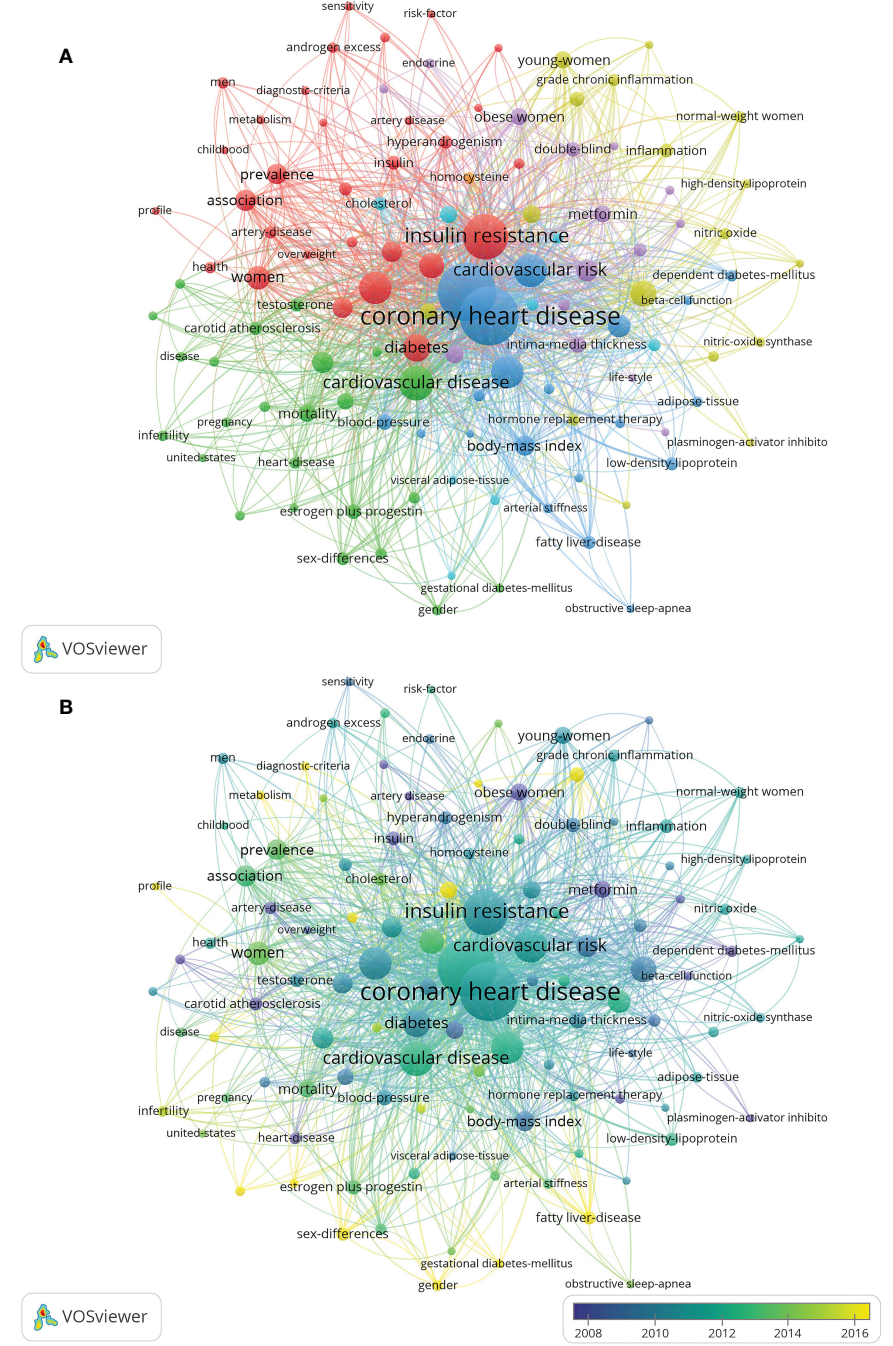
Visualization map of keywords. The minimum number of keyword occurrences threshold is set to 5. Of the 1,491 keywords involved in CHD in PCOS, 115 reached the threshold. **(A)** Network diagram of the keywords. **(B)** Dynamics and trends of the keywords.

A total of 1,491 keywords were extracted from the retrieved records. The minimum number of keyword occurrences threshold is set to 5, and 115 reached the threshold. The network map of the 115 keywords was were showed in **Figure 7A**. **Table 6** listed the top 10 keywords for each cluster. **Table 7** listed the ranking of the top 10 keywords according to their frequency. 4 of the top 10 keywords were clustered in the red cluster. The top 3 keywords were “coronary heart disease (n=1641)”, “polycystic ovary syndrome (n=1614)”, and “insulin-resistance (n=1090)”. **Figure 7B** shows that the hot research directions of CHD and PCOS in recent ten years were oxidative stress, fatty liver-disease, primary prevention, estrogen plus progestin, insulin sensitivity, genome-wide association, metabolism, and dyslipidemia, et al.

**Table 6:** Co-occurrence analysis of keywords and top 10 keywords in the 6 clusters.

Cluster	Color	Keywords (occurences)
Cluster 1	red	insulin resistance (146) risk factors(74) diabetes(53) obesity(45) women(39) association(31) postmenopausal women(30) prevalence(30) term-follow-up(30) insulin(14)
Cluster 2	green	cardiovascular disease(88) myocardial-infarction(31) mortality(22) hypertension(19) estrogen plus progestin(15) sex-differences(13) carotid atherosclerosis(13) testosterone(13) menopause(10) heart-disease(10)
Cluster 3	blue	coronary heart disease(248) polycystic ovary syndrome(241) cardiovascular risk(80) metabolic syndrome(74) type 2 diabetes(36) body-mass index(30) blood-pressure(18) fatty liver-disease(13) low-density-lipoprotein(12) dependent diabetes-mellitus(9)
Cluster 4	yellow	c-reactive protein(48) endothelial function(24) young-women(21) atherosclerosis(20) oxidative stress(15) inflammation(14) grade chronic inflammation(11) hormone replacement therapy(10) nitric oxide(10) normal-weight women(9)
Cluster 5	purple	impaired glucose-tolerance(34) hormone-binding globulin(23) metformin(22) obese women(21) intima-media thickness(19) double-blind(15) plasminogen-activator inhibitor-1(13) weight-loss (9)endocrin (7) estradiol-cyproterone acetate(6)
Cluster 6	indigo	dyslipidemia(22) cholesterol(13) 3rd national-health(11) body-fat distribution(10) density-lipoprotein cholesterol(8) nutrition examination survey(7) visceral adipose-tissue(6) gender differences(5)

**Table 7:** Ranking of keywords usage frequency.

Rank	Keyword	Cluster	Total link strength	Frequency
1	coronary heart disease	3	248	1641
2	polycystic ovary syndrome	3	241	1614
3	insulin resistance	1	146	1090
4	cardiovascular disease	2	88	698
5	cardiovascular risk	3	80	573
6	risk factors	1	74	535
7	metabolic syndrome	3	74	580
8	diabetes	1	53	421
9	c-reactive protein	4	48	360
10	obesity	1	45	361

The keywords were divided into six categories in different colors: (1) the interaction between CHD risk factors and PCOS women; (2) the relationship between cardiovascular disease and female reproductive system hormone secretion; (3) the interaction between CHD and metabolic syndrome; (4) the relationship between c-reactive protein and endothelial function and oxidative stress in PCOS patients; (5) the potential positive effect of metformin on reducing CHD risk factors in PCOS patients; (6) the study of serum cholesterol and body-fat distribution in patients with CHD in PCOS.

**Figure 8:**
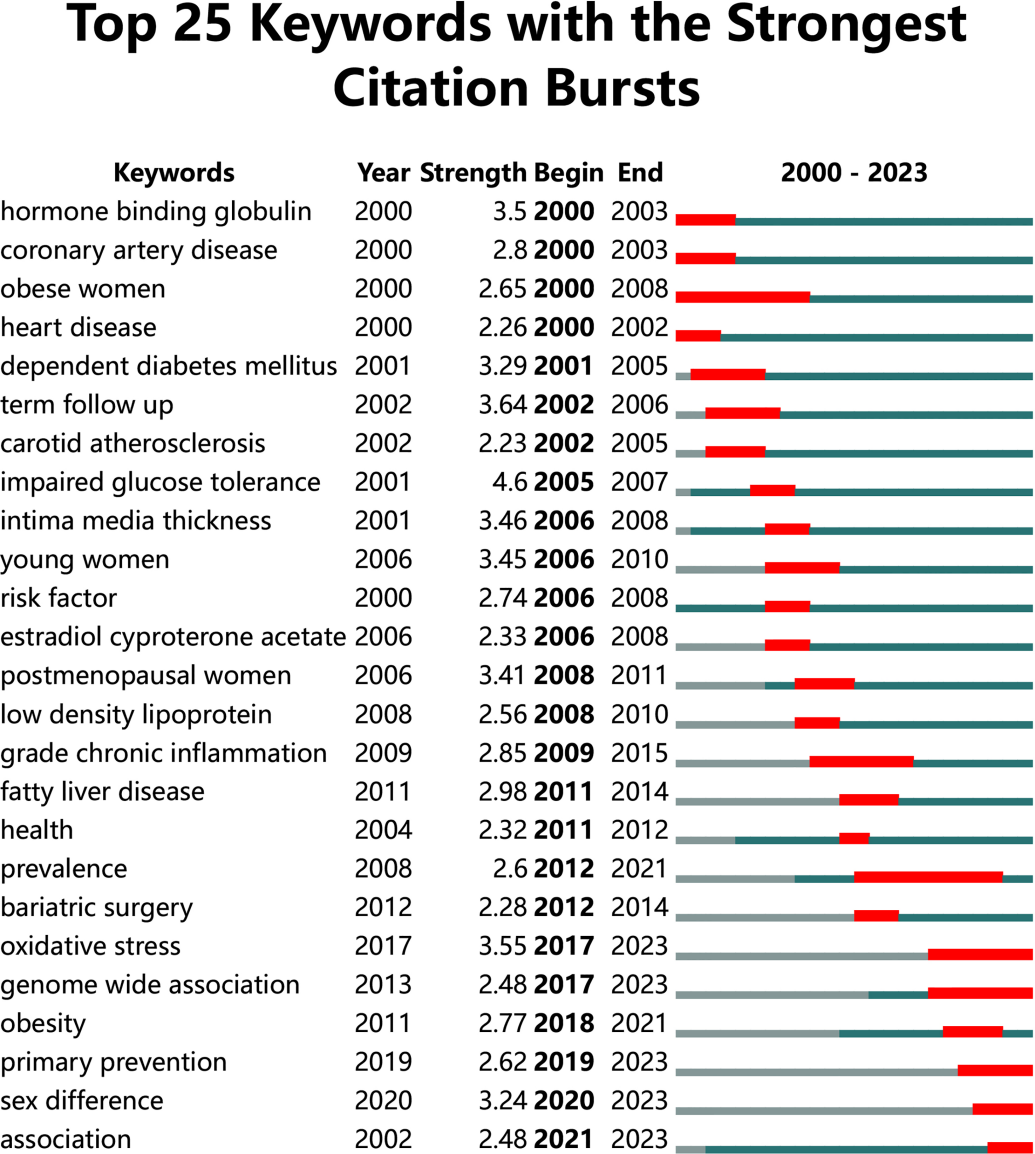
Top 25 keywords with the strongest citation bursts.

Burst words detection results revealed that popular keywords had undergone obvious annual changes (**Figure 8**). In recent five years, the most popular keywords included “oxidative stress”, “genome-wide association”, “obesity”, “primary prevention”, and “sex difference”.

## 4 Discussion

This study conducted a statistical analysis of the literature on the WOS database to retrieve studies on the association between PCOS and CHD and obtained a total of 312 pieces of literature, generally reflecting the trend and hot spot of related research. Its research fields mainly focused on endocrinology metabolism, obstetrics and gynecology, cardiovascular diseases, and reproductive medicine, et al., indicating that the research field was relatively popular. The distribution trend result showed that the literature was on the rise from 2002 to 2008. However, it appeared to be a steady trend in recent years, which reflected that the research on the relationship between CHD and PCOS had entered a relative bottleneck period.

The publication quantity and citation frequency of literature are important indicators to evaluate the scientific research strength of a certain country, region or institution. The country with the most papers was the United States, and the institutions with the most papers were Harvard University, the University of Athens, and Monash University, which showed that the difference in scientific research levels in different countries and regions was considerably large. It was related to the earlier research start, stronger scientific research ability and economic strength in a handful of countries/regions and institutions. Moreover, there were still issues regarding more cooperation among research teams and cross-regional and cross-institutional communication. It is suggested to coordinate research resources from the level of relevant national departments to facilitate cooperation opportunities and further study of different research institutions.

In terms of journals, the Journal of clinical endocrinology & metabolism, Fertility and sterility, and Human reproduction were the three most publishing journals with relevant articles. In addition, after a comprehensive analysis of the authors’ network, Dr. Wang, Ping, Dr. Glueck, Charles J, Dr. Wild, Robert A, Dr. Lambrinoudaki, Irene, and Dr. Azziz, Ricardo ranked in the top five based on the the amount of publications. The researchers and teams involved in relevant studies should pay attention to cooperation and communication with them in future research. The team of Dr. Wang, Ping and Dr. Glueck, Charles J proposed that metformin safely improved the risk factors of CHD and endocrine diseases and promoted the resumption of normal menstruation in the study of the metformin diet in women with PCOS (20). In addition, the metformin diet effectively and safely reduced body weight and LDL-C while increased HDL-C in women with PCOS and kept these outcomes stable over four years (21). Among the 312 pieces of literature cited most frequently, Booth (2012) (15), Pedersen (2015) (16), Moran (2010) (17), Liu (2003) (22), and Wild (2000) (19) et al.ranked the top 5. The most frequently co-cited pieces of literature were Chang (2004) (23), Talbott (1995) (24), Talbott (2000) (25), Dahlgren (1992) (26), and Wild (2000) (19), et al. A comprehensive analysis of co-citation frequency showed that Wild, Talbott and other representatives had higher citation frequency on the whole. Journals with high co-citation frequency were mainly in the field of reproduction and endocrine metabolism.

A keyword is a high summary and refinement of the literature content, which reflects the core substance and value of the full article and responds to hot spots and research frontiers. Six categories were obtained by cluster analysis of keywords. The first cluster emphasized the association between CHD risk factors and PCOS. CHD risk factors include insulin resistance, diabetes and obesity et al. The incidence of cardiovascular disease (including hypertension and dyslipidemia) was higher in PCOS women than in non-PCOS women (27). Young women with PCOS were at increased risk of myocardial infarction, angina and revascularization, but weight and T2DM were modifiable potential risk factors that warranted intervention (6). A study conducted on Chinese women with PCOS showed that serum lipids, glucose, insulin and homeostasis model assessment of insulin resistance (HOMA-IR) levels were higher in the hypertensives group than in the normotensive group after matching for BMI, which indicated that elevated blood pressure was a marker of metabolic risk and should be measured and monitored in PCOS women (28). In particular, a considerable number of young women with a long course of PCOS disease had a significantly increased risk of CHD and should have a long-term health management plan. Even if treatment measures have been taken for the occurrence of cardiovascular disease, continuous or even lifelong follow-up should be conducted. Although there was a strong association between CHD risk factors and PCOS, no evidence suggested that women with PCOS might be affected by an increased risk of cardiovascular death (29).

The second cluster focused on the relationship between cardiovascular disease and female reproductive system hormone secretion. Testosterone promoted visceral fat accumulation and insulin resistance by inhibiting lipolysis and promoting adipogenesis (30). Furthermore, increased visceral fat was directly related to insulin resistance and carotid intima-media thickness (31, 32). According to existing studies, androgen excess and insulin resistance may be responsible for developing all features of metabolic syndrome in PCOS (29). Future studies should investigate in detail the potential role of androgen excess in determining insulin resistance status, particularly in relation to the high risk of developing T2DM.

The third and sixth clusters demonstrated the interaction between CHD and metabolic syndromes such as diabetes-mellitus and dyslipidemia et al. in PCOS patients. Both obese and non-obese PCOS patients had greater visceral fat than non-PCOS controls (33). Also, visceral fat index (VAI) levels were higher in the PCOS group than non-PCOS control after matching for age and BMI (34). PCOS women were more likely to be obese than in non-PCOS controls and proned to have more visceral fat and a higher visceral fat index, which was strongly associated with insulin resistance (34). Furthermore, studies have shown that lipid accumulation (LAP) measurement can help identify a high cardiometabolic risk subgroup in PCOS patients (35). In summary, PCOS was associated with several endocrine and metabolic diseases, including obesity, insulin resistance and diabetes, hypertension, and dyslipidemia, which increased the risk of subclinical cardiovascular disease (1). Although the presence of lipid abnormalities, fibrinolysis abnormalities, and insulin resistance predictably placed PCOS patients at high risk for cardiovascular disease (36), further and more prospective studies should be conducted to evaluate their correlation.

The fourth cluster illustrated the relationship between CRP, endothelial function, and oxidative stress in PCOS. As previously mentioned, PCOS patients had more visceral fat, its adipocyte hypertrophy triggered an inflammatory response, and hyperandrogenemia exacerbated the inflammatory response (37). Moreover, mononuclear cells in adipose tissue were initiated to secrete pro-inflammatory cytokines in response to glucose and saturated fat uptake (38). Jena et al. studied 58 women newly diagnosed with PCOS and found that inflammatory markers were higher in patients with PCOS than in non-PCOS control after matching for age and BMI (33). In addition, insulin resistance, oxidative stress, elevated plasma homocysteine levels, and changes in lipid profiles (a risk factor for cardiovascular disease) were more frequent in PCOS compared with healthy subjects (39). Serum high-sensitive CRP (HS-CRP) levels in patients with PCOS were positively correlated with BMI, LDL, total cholesterol (TC) and triglyceride and negatively correlated with HDL (40). Previous studies had shown a strong correlation between homocysteine and CRP expression in VSMCs, and elevated homocysteine levels induced CRP expression at the transcriptional and translational levels by controlling the n-methyl-D-aspartate receptor (NMDAr) signaling pathway in VSMCs (41, 42). Elevated homocysteine enhanced platelet adhesion to endothelial cells while promoting the production of pre-thrombotic factors such as tissue plasminogen activators and beta-thromboglobulin (43, 44). Therefore, endothelial dysfunction may be one of the multiple mechanisms that may increase the risk of CHD in PCOS, especially by resulting in arterial hypertension, which was strongly associated with disordered levels of reactive oxygen species (ROS) generated in oxidative stress progress (45).

As for the fifth cluster, we concluded the potential positive effect of metformin on reducing CHD risk factors in PCOS patients. A study by Velazquez et al. (46) included 16 non-diabetic PCOS patients who took metformin (1.5 g/d) for eight weeks. The results found that metformin reduced PCOS patients’ levels of the anti-proto plasminogen activator inhibitor type 1 with a 1.3% decrease in BMI. Metformin may be preferred over combined oral contraceptives (COCs) in patients with PCOS since COCs were associated with an increased risk of thrombosis compared to metformin. What’s more, Burchall et al. (47) studied 60 overweight women with PCOS (mean BMI 37 kg/m^2^) who were randomly assigned to the metformin group, high-dose oral COC group, or low-dose COC plus spironolactone group. The results showed that abnormal coagulation events were observed in two COC groups but not in the metformin group. In addition, studies have shown that metformin had an advantage in reducing diastolic blood pressure and triglyceride, thereby predicting favorable metabolic and cardiovascular outcomes in women with PCOS. Moreover, metformin was more effective in reducing hyperandrogenemia indicators (48). However, there were different opinions. A previous study had shown that metformin could promote menstrual recovery in PCOS patients to a certain extent but has no regulating effect on fasting insulin, HOMA-IR, blood lipid, androstenedione and other secretory indicators (49). Carotid intima-media thickness (IMT), ambulatory blood pressure monitoring (ABPM) and other cardiovascular risk factors were not significantly affected (50). More studies are needed to explore the role of metformin in reducing the risk of CHD in PCOS patients.

This study detected 25 burst words that appeared from 2000 to 2023. The burst words reflected the emerging trends and abrupt changes in current research hotspots. According to **Figure 8**, the research hotspots in the recent five years were oxidative stress, genome-wide association, obesity, primary prevention, and sex difference. Since 2017, oxidative stress and genome-wide association have been maintained a high level of attention. Previous literature indicated a significant increase in oxidative stress in PCOS, leading to metabolic dysfunction and cardiovascular disorder features (51, 52). Therefore, the development of prevention and treatment strategies for CHD risk in these patients should involve further research on reducing oxidative stress. With the coming era of big data, genome-wide association study (GWAS) was an effective research strategy for the identification of disease susceptibility genes/loci, which pointed out the direction of PCOS research and laid a solid foundation for personalized diagnosis, prognosis and treatment of PCOS (53–55). Although GWAS provided important clues to the genetic basis of CHD in PCOS, further research is required, such as fine-mapping of susceptibility sites and functional studies of risk genes. Since 2018, burst words gradually turned to obesity and primary prevention, suggesting that the majority of scholars realize the importance of prevention. Also, previous studies suggested that after PCOS was diagnosed in obese women, they should be given primary prevention of CHD during the early stage (56, 57). Moreover, it is worth noting that sex different in the recent three years with a high strength score, and its research duration was shorter than other burst words, indicating the research results were still insufficient and there was less breakthrough research.

Similar to other bibliometrics studies, our research had some shortcomings that must be addressed. Our study’s limitations were as follows: This study only retrieved the most commonly used WOS database, lacking studies on other databases, resulting in some relevant articles may not have been covered. Also, we retrieved articles written in English; thus non-English studies with high quality were not included in our study. Moreover, part of the meta-analysis was recognized as the original article in the WOS database, which may cause discrepancies in the number of reviews and original articles.

## 5 Conclusion

This study explored the relationship between PCOS and CHD and displayed a deep insight into the status and trends of the research field with the bibliometric analysis method. In recent five years, the hotspots of the association between PCOS and CHD were oxidative stress, genome-wide association, obesity, primary prevention, and sex difference. Our study had a certain reference value for further research on the relationship between PCOS and CHD and future relevant research topics hunting.

## Data availability statement

All data generated or analyzed during this study are included in this article. Further inquiries can be directed to the corresponding author.

## Author contributions

XL and HH contributed equally to the research. XL, YW and HH retrieved articles. XL and HH wrote the first draft of the manuscript. HZ and LW performed the data collection. JY provided figures. JF and SF critically revised the paper. SF designed research. JF provided funding support. All authors contributed to the article and approved the submitted version.

## References

[1] Gomez JMD VanHise K Stachenfeld N Chan JL Merz NB Shufelt C. Subclinical cardiovascular disease and polycystic ovary syndrome. Fertil Steril. (2022) 117(5):912–23. doi: 10.1016/j.fertnstert.2022.02.028 PMC10322116 35512975

[2] Sadeghi HM Adeli I Calina D Docea AO Mousavi T Daniali M. Polycystic ovary syndrome: a comprehensive review of pathogenesis, management, and drug repurposing. Int J Mol Sci (2022) 23(2):583–614. doi: 10.3390/ijms23020583 PMC8775814 35054768

[3] Ganie MA Vasudevan V Wani IA Baba MS Arif T Rashid A. Epidemiology, pathogenesis, genetics & management of polycystic ovary syndrome in India. Indian J Med Res (2019) 150(4):333–44. doi: 10.4103/ijmr.IJMR_1937_17 PMC6902362 31823915

[4] Scicchitano P Dentamaro I Carbonara R Bulzis G Dachille A Caputo P. Cardiovascular risk in women with PCOS. Int J Endocrinol Metab (2012) 10(4):611–8. doi: 10.5812/ijem.4020 PMC3693634 23843832

[5] Fauser BC Tarlatzis BC Rebar RW Legro RS Balen AH Lobo R. Consensus on women's health aspects of polycystic ovary syndrome (PCOS): the Amsterdam ESHRE/ASRM-sponsored 3rd PCOS consensus workshop group. Fertil Steril. (2012) 97(1):28–38.e25. doi: 10.1016/j.fertnstert.2011.09.024 22153789

[6] Berni TR Morgan CL Rees DA. Women with polycystic ovary syndrome have an increased risk of major cardiovascular events: a population study. J Clin Endocrinol Metab (2021) 106(9):e3369–e80. doi: 10.1210/clinem/dgab392 PMC8372630 34061968

[7] Rajendran S Willoughby SR Chan WP Liberts EA Heresztyn T Saha M. Polycystic ovary syndrome is associated with severe platelet and endothelial dysfunction in both obese and lean subjects. Atherosclerosis. (2009) 204(2):509–14. doi: 10.1016/j.atherosclerosis.2008.09.010 19027116

[8] Tosi F Dorizzi R Castello R Maffeis C Spiazzi G Zoppini G. Body fat and insulin resistance independently predict increased serum c-reactive protein in hyperandrogenic women with polycystic ovary syndrome. Eur J Endocrinol (2009) 161(5):737–45. doi: 10.1530/EJE-09-0379 19713424

[9] Guzelmeric K Alkan N Pirimoglu M Unal O Turan C. Chronic inflammation and elevated homocysteine levels are associated with increased body mass index in women with polycystic ovary syndrome. Gynecol Endocrinol (2007) 23(9):505–10. doi: 10.1080/09513590701554306 17852421

[10] Wild RA. Polycystic ovary syndrome: a risk for coronary artery disease? Am J Obstet Gynecol (2002) 186(1):35–43. doi: 10.1067/mob.2002.119180 11810081

[11] Anderson SA Barry JA Hardiman PJ. Risk of coronary heart disease and risk of stroke in women with polycystic ovary syndrome: a systematic review and meta-analysis. Int J Cardiol (2014) 176(2):486–7. doi: 10.1016/j.ijcard.2014.06.079 25096551

[12] Guan C Zahid S Minhas AS Ouyang P Vaught A Baker VL. Polycystic ovary syndrome: a "risk-enhancing" factor for cardiovascular disease. Fertil Steril. (2022) 117(5):924–35. doi: 10.1016/j.fertnstert.2022.03.009 35512976

[13] van Eck NJ Waltman L. Software survey: VOSviewer, a computer program for bibliometric mapping. Scientometrics. (2010) 84(2):523–38. doi: 10.1007/s11192-009-0146-3 PMC2883932 20585380

[14] Synnestvedt MB Chen C Holmes JH. CiteSpace II: visualization and knowledge discovery in bibliographic databases. AMIA Annu Symp Proc (2005) 2005:724–8. doi: 10.2196/27434 PMC1560567 16779135

[15] Booth FW Roberts CK Laye MJ. Lack of exercise is a major cause of chronic diseases. Compr Physiol (2012) 2(2):1143–211. doi: 10.1002/cphy.c110025 PMC4241367 23798298

[16] Pedersen BK Saltin B. Exercise as medicine - evidence for prescribing exercise as therapy in 26 different chronic diseases. Scand J Med Sci Sports. (2015) 25 Suppl 3:1–72. doi: 10.1111/sms.12581 26606383

[17] Moran LJ Misso ML Wild RA Norman RJ. Impaired glucose tolerance, type 2 diabetes and metabolic syndrome in polycystic ovary syndrome: a systematic review and meta-analysis. Hum Reprod Update. (2010) 16(4):347–63. doi: 10.1093/humupd/dmq001 20159883

[18] Yeap BB. Androgens and cardiovascular disease. Curr Opin Endocrinol Diabetes Obes (2010) 17(3):269–76. doi: 10.1097/MED.0b013e3283383031 20186051

[19] Wild S Pierpoint T McKeigue P Jacobs H. Cardiovascular disease in women with polycystic ovary syndrome at long-term follow-up: a retrospective cohort study. Clin Endocrinol (Oxf). (2000) 52(5):595–600. doi: 10.1046/j.1365-2265.2000.01000.x 10792339

[20] Morrison JA Glueck CJ Daniels SR Horn PS Wang P. Determinants of ApoB, ApoA1, and the ApoB/ApoA1 ratio in healthy schoolgirls, prospectively studied from mean ages 10 to 19 years: the Cincinnati national growth and health study. Metabolism. (2012) 61(10):1377–87. doi: 10.1016/j.metabol.2012.02.014 PMC3752903 22512822

[21] Glueck CJ Aregawi D Agloria M Winiarska M Sieve L Wang P. Sustainability of 8% weight loss, reduction of insulin resistance, and amelioration of atherogenic-metabolic risk factors over 4 years by metformin-diet in women with polycystic ovary syndrome. Metabolism. (2006) 55(12):1582–9. doi: 10.1016/j.metabol.2006.08.001 17142128

[22] Liu PY Death AK Handelsman DJ. Androgens and cardiovascular disease. Endocr Rev (2003) 24(3):313–40. doi: 10.1210/er.2003-0005 12788802

[23] Rotterdam EA-SPCWG. Revised 2003 consensus on diagnostic criteria and long-term health risks related to polycystic ovary syndrome. Fertil Steril. (2004) 81(1):19–25. doi: 10.1016/j.fertnstert.2003.10.004 14711538

[24] Talbott E Guzick D Clerici A Berga S Detre K Weimer K. Coronary heart disease risk factors in women with polycystic ovary syndrome. Arterioscler Thromb Vasc Biol (1995) 15(7):821–6. doi: 10.1161/01.atv.15.7.821 7600112

[25] Talbott EO Guzick DS Sutton-Tyrrell K McHugh-Pemu KP Zborowski JV Remsberg KE. Evidence for association between polycystic ovary syndrome and premature carotid atherosclerosis in middle-aged women. Arterioscler Thromb Vasc Biol (2000) 20(11):2414–21. doi: 10.1161/01.atv.20.11.2414 11073846

[26] Dahlgren E Janson PO Johansson S Lapidus L Oden A. Polycystic ovary syndrome and risk for myocardial infarction. evaluated from a risk factor model based on a prospective population study of women. Acta Obstet Gynecol Scand (1992) 71(8):599–604. doi: 10.3109/00016349209006227 1336918

[27] Glintborg D Rubin KH Nybo M Abrahamsen B Andersen M. Cardiovascular disease in a nationwide population of Danish women with polycystic ovary syndrome. Cardiovasc Diabetol (2018) 17(1):37. doi: 10.1186/s12933-018-0680-5 29519249 PMC5844097

[28] Shi Y Cui Y Sun X Ma G Ma Z Gao Q. Hypertension in women with polycystic ovary syndrome: prevalence and associated cardiovascular risk factors. Eur J Obstet Gynecol Reprod Biol (2014) 173:66–70. doi: 10.1016/j.ejogrb.2013.11.011 24368020

[29] Pasquali R. Metabolic syndrome in polycystic ovary syndrome. Front Horm Res (2018) 49:114–30. doi: 10.1159/000485995 29894990

[30] Rosenfield RL Ehrmann DA. The pathogenesis of polycystic ovary syndrome (PCOS): the hypothesis of PCOS as functional ovarian hyperandrogenism revisited. Endocr Rev (2016) 37(5):467–520. doi: 10.1210/er.2015-1104 27459230 PMC5045492

[31] Tripathy P Sahu A Sahu M Nagy A. Ultrasonographic evaluation of intra-abdominal fat distribution and study of its influence on subclinical atherosclerosis in women with polycystic ovarian syndrome. Eur J Obstet Gynecol Reprod Biol (2017) 217:18–22. doi: 10.1016/j.ejogrb.2017.08.011 28850821

[32] Cascella T Palomba S De Sio I Manguso F Giallauria F De Simone B. Visceral fat is associated with cardiovascular risk in women with polycystic ovary syndrome. Hum Reprod (2008) 23(1):153–9. doi: 10.1093/humrep/dem356 18024952

[33] Jena D Choudhury AK Mangaraj S Singh M Mohanty BK Baliarsinha AK. Study of visceral and subcutaneous abdominal fat thickness and its correlation with cardiometabolic risk factors and hormonal parameters in polycystic ovary syndrome. Indian J Endocrinol Metab (2018) 22(3):321–7. doi: 10.4103/ijem.IJEM_646_17 PMC6063187 30090722

[34] Durmus U Duran C Ecirli S. Visceral adiposity index levels in overweight and/or obese, and non-obese patients with polycystic ovary syndrome and its relationship with metabolic and inflammatory parameters. J Endocrinol Invest. (2017) 40(5):487–97. doi: 10.1007/s40618-016-0582-x 27838846

[35] Kaluzna M Czlapka-Matyasik M Bykowska-Derda A Moczko J Ruchala M Ziemnicka K. Indirect predictors of visceral adipose tissue in women with polycystic ovary syndrome: a comparison of methods. Nutrients. (2021) 13(8):2494–506. doi: 10.3390/nu13082494 PMC8401513 34444654

[36] Dunaif A. Insulin resistance and the polycystic ovary syndrome: mechanism and implications for pathogenesis. Endocr Rev (1997) 18(6):774–800. doi: 10.1210/edrv.18.6.0318 9408743

[37] Rosen ED Spiegelman BM. What we talk about when we talk about fat. Cell (2014) 156(1-2):20–44. doi: 10.1016/j.cell.2013.12.012 24439368 PMC3934003

[38] Gonzalez F. Nutrient-induced inflammation in polycystic ovary syndrome: role in the development of metabolic aberration and ovarian dysfunction. Semin Reprod Med (2015) 33(4):276–86. doi: 10.1055/s-0035-1554918 26132932

[39] Yilmaz M Bukan N Ayvaz G Karakoc A Toruner F Cakir N. The effects of rosiglitazone and metformin on oxidative stress and homocysteine levels in lean patients with polycystic ovary syndrome. Hum Reprod (2005) 20(12):3333–40. doi: 10.1093/humrep/dei258 16123091

[40] Verit FF. High sensitive serum c-reactive protein and its relationship with other cardiovascular risk factors in normoinsulinemic polycystic ovary patients without metabolic syndrome. Arch Gynecol Obstet. (2010) 281(6):1009–14. doi: 10.1007/s00404-009-1226-6 19771438

[41] Pang X Liu J Zhao J Mao J Zhang X Feng L. Homocysteine induces the expression of c-reactive protein *via* NMDAr-ROS-MAPK-NF-kappaB signal pathway in rat vascular smooth muscle cells. Atherosclerosis. (2014) 236(1):73–81. doi: 10.1016/j.atherosclerosis.2014.06.021 25016361

[42] Li Y Zhao Q Cao Y Si J Li J Cao K. Probucol decreases homocysteine-stimulated CRP production in rat aortic smooth muscle cells *via* regulating HO-1/NADPH oxidase/ROS/p38 pathway. Acta Biochim Biophys Sin (Shanghai). (2021) 53(2):212–9. doi: 10.1093/abbs/gmaa163 33382068

[43] Chen L Wang B Wang J Ban Q Wu H Song Y. Association between serum total homocysteine and arterial stiffness in adults: a community-based study. J Clin Hypertens (Greenwich). (2018) 20(4):686–93. doi: 10.1111/jch.13246 PMC8031326 29481715

[44] Faeh D Chiolero A Paccaud F. Homocysteine as a risk factor for cardiovascular disease: should we (still) worry about? Swiss Med Wkly (2006) 136(47-48):745–56. doi: 10.4414/smw.2006.11283 17225194

[45] Duica F Danila CA Boboc AE Antoniadis P Condrat CE Onciul S. Impact of increased oxidative stress on cardiovascular diseases in women with polycystic ovary syndrome. Front Endocrinol (Lausanne) (2021) 12:614679. doi: 10.3389/fendo.2021.614679 33679617 PMC7930620

[46] Velazquez EM Mendoza SG Wang P Glueck CJ. Metformin therapy is associated with a decrease in plasma plasminogen activator inhibitor-1, lipoprotein(a), and immunoreactive insulin levels in patients with the polycystic ovary syndrome. Metabolism. (1997) 46(4):454–7. doi: 10.1016/S0026-0495(97)90066-4 9109854

[47] Burchall GF Piva TJ Ranasinha S Teede HJ. Differential effects on haemostatic markers by metformin and the contraceptive pill: a randomized comparative trial in PCOS. Thromb Haemost. (2017) 117(11):2053–62. doi: 10.1160/TH17-04-0248 29202210

[48] Soldat-Stankovic V Popovic Pejicic S Stankovic S Jovanic J Bjekic-Macut J Livadas S. The effect of myoinositol and metformin on cardiovascular risk factors in women with polycystic ovary syndrome: a randomized controlled trial. Acta Endocrinol (Buchar). (2021) 17(2):241–7. doi: 10.4183/aeb.2021.241 PMC8665246 34925574

[49] Guan Y Wang D Bu H Zhao T Wang H. The effect of metformin on polycystic ovary syndrome in overweight women: a systematic review and meta-analysis of randomized controlled trials. Int J Endocrinol (2020) 2020:5150684. doi: 10.1155/2020/5150684 33014044 PMC7519180

[50] Sahin Y Unluhizarci K Yilmazsoy A Yikilmaz A Aygen E Kelestimur F. The effects of metformin on metabolic and cardiovascular risk factors in nonobese women with polycystic ovary syndrome. Clin Endocrinol (Oxf). (2007) 67(6):904–8. doi: 10.1111/j.1365-2265.2007.02985.x 17666089

[51] Rudnicka E Duszewska AM Kucharski M Tyczynski P Smolarczyk R. OXIDATIVE STRESS AND REPRODUCTIVE FUNCTION: oxidative stress in polycystic ovary syndrome. Reproduction (2022) 164(6):F145–F54. doi: 10.1530/REP-22-0152 36279177

[52] Papalou O Victor VM Diamanti-Kandarakis E. Oxidative stress in polycystic ovary syndrome. Curr Pharm Des (2016) 22(18):2709–22. doi: 10.2174/1381612822666160216151852 26881435

[53] Brower MA Hai Y Jones MR Guo X Chen YI Rotter JI. Bidirectional mendelian randomization to explore the causal relationships between body mass index and polycystic ovary syndrome. Hum Reprod (2019) 34(1):127–36. doi: 10.1093/humrep/dey343 PMC6295958 30496407

[54] Day F Karaderi T Jones MR Meun C He C Drong A. Large-Scale genome-wide meta-analysis of polycystic ovary syndrome suggests shared genetic architecture for different diagnosis criteria. PLoS Genet (2018) 14(12):e1007813. doi: 10.1371/journal.pgen.1007813 30566500 PMC6300389

[55] Day FR Hinds DA Tung JY Stolk L Styrkarsdottir U Saxena R. Causal mechanisms and balancing selection inferred from genetic associations with polycystic ovary syndrome. Nat Commun (2015) 6:8464. doi: 10.1038/ncomms9464 26416764 PMC4598835

[56] Geraghty L Figtree GA Schutte AE Patel S Woodward M Arnott C. Cardiovascular disease in women: from pathophysiology to novel and emerging risk factors. Heart Lung Circ (2021) 30(1):9–17. doi: 10.1016/j.hlc.2020.05.108 32843293

[57] Christian RC Dumesic DA Behrenbeck T Oberg AL Sheedy PF Fitzpatrick LA. Prevalence and predictors of coronary artery calcification in women with polycystic ovary syndrome. J Clin Endocrinol Metab (2003) 88(6):2562–8. doi: 10.1210/jc.2003-030334 12788855

